# Age of First Overweight and Obesity, COVID-19 and Long COVID in Two British Birth Cohorts

**DOI:** 10.1007/s44197-023-00093-5

**Published:** 2023-02-22

**Authors:** Charis Bridger Staatz, David Bann, George B. Ploubidis, Alissa Goodman, Richard J. Silverwood

**Affiliations:** Centre for Longitudinal Studies, UCL Social Research Institute, University College London, London, UK

**Keywords:** Body mass index, Obesity, COVID-19, Long COVID, COVID-19 severity, Longitudinal

## Abstract

Longer exposure to obesity, and thus a longer period in an inflamed state, may increase susceptibility to infectious diseases and worsen severity. Previous cross-sectional work finds higher BMI is related to worse COVID-19 outcomes, but less is known about associations with BMI across adulthood. To examine this, we used body mass index (BMI) collected through adulthood in the 1958 National Child Development Study (NCDS) and the 1970 British Cohort Study (BCS70). Participants were grouped by the age they were first overweight (> 25 kg/m^2^) and obese (> 30 kg/m^2^). Logistic regression was used to assess associations with COVID-19 (self-reported and serology-confirmed), severity (hospital admission and contact with health services) and long-COVID reported at ages 62 (NCDS) and 50 (BCS70). An earlier age of obesity and overweight, compared to those who never became obese or overweight, was associated with increased odds of adverse COVID-19 outcomes, but results were mixed and often underpowered. Those with early exposure to obesity were over twice as likely in NCDS (odds ratio (OR) 2.15, 95% confidence interval (CI) 1.17–4.00) and three times as likely in BCS70 (OR 3.01, 95% CI 1.74–5.22) to have long COVID. In NCDS they were also over four times as likely to be admitted to hospital (OR 4.69, 95% CI 1.64–13.39). Most associations were somewhat explained by contemporaneous BMI or reported health, diabetes or hypertension; however, the association with hospital admission in NCDS remained. An earlier age of obesity onset is related to COVID-19 outcomes in later life, providing evidence of the long-term impact of raised BMI on infectious disease outcomes in midlife.

**Supplementary Information:**

The online version contains supplementary material available at 10.1007/s44197-023-00093-5.

## Background

SARS-CoV-2 is a novel respiratory virus that predominantly attacks the respiratory system but also impacts other systems, such as cardiovascular health [1]. A growing body of evidence has documented the long-term consequences of COVID-19, commonly referred to as (Sect. 2.3.3) [2, 3]. However, the type and number of symptoms experienced by long COVID sufferers can vary drastically between studies [3, 4], and thus the different definitions used influences reporting of the prevalence of long COVID.

In an analysis of 10 longitudinal studies in the UK, the prevalence of long COVID was as high as 17% among middle-aged adults and using a definition of long COVID that considered all symptoms lasting more than 12 weeks, irrespective of severity, but only 4.8–5.4% when considering symptoms that limited day-to-day function [5]. In a study of over 500,000 adults in the UK, a third of individuals had at least one persistent symptom out of 29 possible symptoms after 12 weeks following a SARS-CoV-2 infection [2]. Recent work has found that long-term symptoms after acute COVID-19 are similar to other respiratory illnesses [6]. The Office of National Statistics reported that 3.3% of the UK population had self-reported long COVID [7], using the National Institute for Health and Care Excellence (NICE) definition of symptoms lasting longer than 4 weeks [8].

Obesity is excess adiposity that increases the risk of disease [9], and impacts the cardiovascular [10], respiratory [11] and immune systems in the body [12]. Research has found associations between higher BMI and increased risk of contracting SARS-CoV-2 [13, 14] and increased severity of illness [14, 15]. A systematic review and meta-analysis showed that individuals who were severely obese were 2.5 times more likely to be hospitalised because of COVID-19 and twice as likely to die compared to those of normal weight, although the strength of association weakened from 2020 to 2021 as the pandemic progressed [15]. There is emerging evidence that having a higher BMI may also be related to an increased risk of long COVID [5, 16, 17]. In an analysis of 10 longitudinal studies and electronic health records for 1.1 million people in the UK, individuals reporting overweight/obesity at the most recent pre-pandemic measure were 24–31% more likely to report COVID-19 symptoms lasting more than 4 weeks, compared to those of healthy weight [5].

Previous research has predominantly focused on cross-sectional associations or used a single measure of obesity directly prior to the pandemic, but fewer studies have looked at longitudinal associations between life course measures of BMI and SARS-CoV-2 infection, COVID-19 severity, and long COVID in mid-life. Research has shown that an earlier age at which individuals first become overweight, measured by BMI, is related to increased risk of other health outcomes in later life, such as chronic kidney diseases [18], cardiovascular disease [19], high blood pressure [20, 21], cholesterol and diabetes [21]. Upward movement of weight categories is more common across adulthood than downward movement, which is comparably rare [22, 23]. Therefore, once someone becomes overweight or obese, they are likely to remain so. Obesity is considered a state of low-grade inflammation [24, 25], and long periods of inflammation are harmful to health with consequences for the immune and cardiovascular systems [24, 25]. It is hypothesised that becoming overweight and obese earlier in life may therefore increase the risk of severe COVID-19 outcomes, compared to individuals who are never overweight or obese. Moreover, as BMI tracks across the life course and increases the risk of chronic diseases, BMI at younger ages may be related to COVID-19 severity through higher achieved BMI and the presence of chronic diseases in later adulthood.

Associations between earlier age of overweight and obesity and increased risk of severe COVID-19 outcomes will also likely differ by cohort and age groups. Previous research has found COVID-19 outcomes to be more severe among older populations [26], and among 10 longitudinal studies there was a 3% rise in reporting of functionally limiting COVID-19 symptoms lasting > 4 weeks for each decade increase in age [5]. As earlier-born cohorts would be older at the time of the pandemic, it is possible associations may be more apparent between BMI across adulthood and COVID-19 outcomes. However, more recently born, and, therefore, younger, cohorts have typically been less healthy than those that have proceeded them [27], and BMI has been higher across adulthood [28]. It is therefore possible the strength of association between early age of overweight and obesity and severe COVID-19 outcomes may be stronger in adults who were born more recently, where sustained inflammation as a result of higher BMI, may be greater.

Therefore, we aim to estimate the risk of severe COVID-19 outcomes, including long COVID, associated with the age at which participants first become overweight or obese, compared to those who are never overweight or obese, respectively. We utilise the longitudinal nature of two British birth cohorts, the 1958 National Child Development Study (NCDS) and the 1970 British Cohort Study (BCS70), to allow comparison between generations. To explore potential mechanisms of observed associations, secondary analysis additionally explored whether associations between BMI across adulthood and COVID-19, severity and long COVID in mid-life are mediated by BMI, self-reported health and chronic conditions in adulthood.

## Methods

### The Datasets

The NCDS [29] and BCS70 [30] are two British birth cohort studies that have tracked cohort members since infancy. Both cohorts have followed roughly 17,000 individuals, sampled from one week in March in 1958 (NCDS) and one week in April 1970 (BCS70). The cohort members have been followed up 11 (NCDS) and 10 (BCS70) times since birth, with the most recently completed pre-COVID-19 data collection taking place in 2013 for NCDS, and 2016 for BCS70, when cohort members were aged 55 and 46, respectively.

Additionally to the main surveys, NCDS and BCS70 cohort members participated in three waves of data collection during the COVID-19 pandemic when they were age 62 (NCDS) and 50 (BCS70) [31]. The COVID-19 surveys were conducted from May 2020, September to October 2020 and February to March 2021. Response to the COVID-19 surveys increased across waves. In NCDS, 5178 cohort members responded at the first wave, 6282 responded at wave 2, and 6809 responded at wave 3. The equivalent figures in BCS70 were 4223, 5320 and 5758. A total of 7769 NCDS cohort members and 7168 BCS70 cohort members responded to at least one wave of the COVID-19 survey [31].

Cohort members who responded to any of the three COVID-19 surveys were invited to complete a finger prick blood test in March of 2021 allowing for the detection of SARS-CoV-2 antibodies. A total of 3222 NCDS cohort members and 2547 BCS70 cohort members returned a blood sample [32].

### Age First Overweight or Obese

In the NCDS, self-reported weight and height in adulthood were collected at ages 23, 33, 42, 44, 50, and 55, and nurse-measured height and weight were additionally obtained at age 44. In BCS70, self-report weight and height during adulthood were collected at ages 26, 29, 34, 42 and 46, and nurse-measured height and weight were also available at age 46. As height is likely to stay consistent throughout adulthood, a measure for lifetime height was derived as the earliest recorded height in adulthood (i.e. from age 23 onwards in NCDS and age 26 onwards in BCS70).

BMI was used as a proxy for adiposity and was derived for each age by taking weight in kg and dividing it by lifetime height in meters squared (kg/m^2^). The ages at which an individual was first overweight and first obese were derived as the first age at which they had a BMI greater or equal to 25.0 kg/m^2^ and greater or equal to 30 kg/m^2^, respectively.

Where low counts (*N* < 10) were observed in cross tabulations with the outcomes, BMI groups were collapsed to those first overweight or obese at age “23/33”, “42/44”, “50/55” and “never” in the NCDS, and “26/29” “34/42” and “46/never” for BCS70. It was assumed that once cohort members became overweight or obese, they were likely to remain so.

It was not possible to use the age of first overweight or obese as the exposure in mediation analysis, as it overlapped temporally with the mediators. Therefore, a continuous measure of BMI at each age was used.

### COVID-19 Outcomes

#### Self-Reported COVID-19 and Serology-Confirmed SARS-CoV-2

Self-reported COVID-19 illness and infection from the SARS-CoV-2 virus were collected in all three waves of COVID-19 data collection and combined across sweeps to improve statistical power. Because of the wording of the questionnaire which asked “Do you think that you have or had Coronavirus?” and included confirmation through self-reported positive test, both infection from the SARS-CoV-2 virus and COVID-19 illness was likely captured, but we will refer to this as “self-reported COVID-19”. Cohort members reported whether they: had not had a COVID-19 illness; were unsure if they’d had COVID-19 illness; had a strong personal suspicion they’d had COVID-19 illness or had been told by a medical professional that it was likely they had COVID-19 illness; or had received a positive test confirming infection with SARS-CoV-2. Those reporting that they were unsure (NCDS 9.8%, BCS70 13.1%) were grouped with those reporting no COVID-19 illness; the remaining responses were classified as self-reported COVID-19 illness.

Blood samples were returned by cohort members between 21 April and 2 July 2021. Antibody testing was conducted to identify evidence of previous SARS-CoV-2 infection. A binary result was returned, with a positive test (signifying the presence of antibodies against SARS-CoV-2 nucleocapsid (N) protein exceeding the pre-determined threshold), indicating a likely exposure to SARS-CoV-2 infection [32]. Sensitivity and specificity for the immunoassay across populations in Europe, tested > 14 days following a SARS-CoV-2 polymerase chain reaction test, were found to be 97.92% and 99.95%, respectively [33].

#### COVID-19 Severity: Hospital Admission and NHS 111 Contact

At each COVID-19 survey wave, cohort members were asked if they had ever been admitted to the hospital with COVID-19 symptoms (yes or no) and if they had ever contacted NHS 111 or NHS 24 h because of their symptoms (yes or no). For both outcomes, responses were combined across COVID-19 survey waves 1–3 to maintain an adequate sample size and to prevent low counts in cross tabulations with age-first overweight or obese groups. Cohort members that responded “yes” in any one or more sweeps, were classified as having had a hospital admission or contact with NHS 111, respectively.

#### Long COVID

Cohort members who had experienced COVID-19 illness were asked about how long their symptoms prevented them from functioning as normal in the third COVID-19 survey. Those reporting functional limitations for 4 or more weeks were considered as having long COVID, in line with NICE guidance [8]. Individuals who reported having COVID-19 in the same month in which they responded to the question on the length of symptoms were excluded from long COVID analyses to ensure sufficient follow-up time for long COVID to be reliably assessed.

### Potential Confounders

Sex, social class at birth, childhood housing tenure and cognitive ability were included as potential confounders based on previously observed or hypothesised associations with the exposures and outcomes of interest (Figure S1, Supplementary Material). Social class at birth and housing tenure are considered markers of socioeconomic position, which has an established influence on health outcomes including BMI [34] and COVID-19 [35]. Similarly, cognitive ability was also included as a confounder, as it is related to morbidity [36], such as BMI and COVID-19, through SEP and health literacy—the ability to understand and implement public health messaging.

Sex was reported at birth, supplemented by sex reported in the COVID-19 surveys for a small number of BCS70 cohort members where the former was missing. Social class at birth was taken from fathers’ occupational class, using the register generals’ groupings of (1) Other/single parent—not working; (2) V unskilled; (3) IV partly-skilled; (4) III manual; (5) III non-manual; (6) II managerial and technical; (7) I professional. In the NCDS, the groups of “4) III manual” and “5) III non-manual” were reported together as one group, and the “other group” additionally included fathers who were students, retired, sick, deceased or absent. Housing tenure was considered at age 7 in NCDS and age 5 in BCS70: “Owner occupied”, “Council rented”, “Private rented” or “Other/rent free” in NCDS; “Owned outright”, “Being bought”, “Council rented”, “Private rented” or “Tied to occupation/other” in BCS70. Cognitive ability was derived in both cohorts by standardising the first component from a principal component analysis. The components were derived from general ability, reading comprehension, mathematics and copying design test score in NCDS at age 11, and from pictorial language, friendly maths, Edinburgh reading tests and word definitions, similarities, digits and matrices basic ability scores at age 10 in BCS70.

### Mediators

Mediators included were BMI, blood pressure, diabetes and self-rated general health taken at the latest age recorded prior to the pandemic (NCDS: age 55; BCS70: age 46). In both cohorts, participants were asked if they had suffered from diabetes or high blood pressure since their last interview (yes or no). In NCDS participants rated their own general health as either “excellent”, “very good”, “good”, “fair” or “poor”. In BCS70 cohort members indicated if they agreed that their health was excellent by answering “definitely true”, “mostly true”, “do not know”, “mostly false” or “definitely false”.

### Analysis

#### Statistical Analysis

For each outcome, separate logistic regression models were fitted with the first age of overweight or first age obese as the exposure, with never overweight or obese as the reference categories. For collapsed groups in BCS70, “first overweight at age 46/never overweight” was used as the reference category. Both unadjusted and adjusted models including sex, social class at birth, childhood housing tenure and cognitive ability were fitted (Figure S1, Supplementary Material). For the outcome long COVID, individuals not self-reporting previous COVID-19 were excluded. No complete case analysis was presented as approaches to derive the first overweight and obese variables resulted in substantial loss of power or probable misclassification due to incomplete information on BMI across adulthood.

There was likely misclassification of self-report COVID-19, as cohort members who only responded to the first two waves may have been incorrectly classified as not having had COVID-19 if they then went on to have it at the time of the third wave. Therefore, sensitivity analysis was conducted using self-reported COVID-19 at wave 3 only, to explore the impact of probable misclassification of the outcome. This was not done for hospital admission or NHS 111 as numbers were too small when only using wave 3.

#### Secondary Analysis: Exploring Potential Mediators

Mediation analysis was conducted for outcomes where associations with age at first overweight or obese were identified in the main analysis (*p* < 0.1), using a continuous measure of BMI at the equivalent age. We used BMI from a single time point, as opposed to the categorical variable used in the primary analysis that captured the overweight and obesity history across adulthood, to ensure BMI causally proceeded all the mediators. Odds ratios were rescaled so that they represent the change in odds of COVID-19 outcomes per 5 kg/m^2^ increase in BMI. This was done to reflect the difference in BMI between consecutive conventional BMI categories (20–25 kg/m^2^, 25–30 kg/m^2^, etc.) and allow the identification of subtle changes in the odds ratios with the addition of mediators.

Model 1 included adjustment for sex, social class at birth, childhood housing tenure and cognitive ability. Where an association was observed, mediators were included separately in additional models. In these models, effect estimates can be interpreted under certain assumptions as the direct effect of BMI on COVID-19 outcomes, not going through mediators. Firstly, BMI in later life was included (Model 2) to explore if associations with BMI across adulthood were explained through later-life adult BMI. Additional models made adjustments for diabetes (model 3), blood pressure (model 4) and self-rated health (Model 5) to test if associations were mediated through poorer health in later adulthood. Model 6 included diabetes and blood pressure together in a single model, and Model 7 included all four mediators.

#### Missing Data Handling

Missing data patterns were explored (Table S1, Supplementary Material) and missing exposure, covariate and mediator data were handled using multiple imputation [37], to maintain power and reduce bias related to missing data, under the assumption of missing at random [38, 39]. Imputation models were run separately for each outcome obtaining 15 imputations each and included the outcome of interest, lifetime height, weight at each age, all covariates and auxiliary variables. Further details of imputation methods and selected auxiliary variables are provided in the supplementary material (Methods S1).

#### COVID-19 Survey Weights and Inverse Probability Weighting

Multiple imputations addressed item non-response by imputing exposures and covariates to the sub-sample that responded to the COVID-19 surveys. However, as those who responded to the COVID-19 surveys were only a subset of those that respond to the main survey collections, weights deposited with the COVID-19 survey data were used to ensure analyses were representative of the full cohort samples [31]. For analysis using long COVID as the outcome, additional inverse probability (IP) weights were derived to ensure the data were representative of all participants who responded to the COVID-19 surveys, and not just those that had a self-reported COVID-19 illness. This was done to address index event bias [40], which happens when inclusion in the analysis is contingent on the occurrence of a prior event (in this case, self-reported COVID-19). As certain risk factors are likely related to both outcomes (COVID-19 illness and development of long COVID) a naive analysis selecting on self-reported COVID-19 would be effectively conditioning a collider. This could induce spurious associations between shared risk factors, potentially biasing associations with long COVID. The weighting approach employed here was designed to reduce such bias. Further details of how weights were implemented are presented in the supplementary material (Methods S2). Sensitivity analysis was run comparing unweighted analysis to explore the impact of including COVID-19 and IP weights and is reported in the supplementary material.

## Results

### Descriptive Results

Table 1 shows the distribution of the selected covariates and outcomes in NCDS and BCS70. A higher percentage of cohort members reported suspected COVID-19 (15.6% vs. 10.5%) and had serology-confirmed SARS-CoV-2 infection (11.2% vs. 8.4%) in BCS70 compared to NCDS, whilst more cohort members reported long COVID (18.7% vs. 14.8%) in NCDS. A similar proportion in both cohorts reported contacting NHS 24 h, NHS 111 and being admitted to the hospital because of their symptoms.

**Table 1:** Descriptive Table of Covariates, Mediators and Outcomes in BCS70 and NCDS

	NCDS		BCS70	
	*N*	%/mean (SE)	*N*	%/mean (SE)
Sex				
Male	3715	47.8	3168	44.2
Female	4054	52.2	3995	55.8
Total	7769	100.0	7163	100.0
Social class at birth				
Other/single parent—not working	324	4.4	26	0.4
V unskilled	482	6.5	272	4.1
IV partly-skilled	766	10.4	937	14.1
III manual	4250	57.7	2857	43.0
III non manual			1035	15.6
II managerial and technical	1123	15.2	1058	15.9
I professional	423	5.7	454	6.8
Total	7368	100.0	6639	100.0
Housing tenure age 7/5^A^				
Owner occupied	3253	47.8	811	13.8
Being Bought	–	–	2929	49.8
Council rented	2398	35.3	1525	25.9
Private rented	780	11.5	320	5.4
Tied to occupation/rent free/other	372	5.5	297	5.1
Total	6803	100.0	5882	100.0
Standardised cognitive ability age 11/10				
Total	6727	0.50 (0.02)	5208	0.57 (0.03)
Self-rated health age 55/46^B^				
Excellent/definitely true	1030	14.6	1101	18.9
Very good/mostly true	2593	36.7	2913	49.9
Good/do not know	2248	31.8	623	10.7
Fair/mostly false	904	12.8	682	11.7
Poor/definitely false	296	4.2	515	8.8
Total	7071	100.0	5834	100.0
Diabetes 55/46				
No	6669	94.5	5898	96.6
Yes	392	5.6	208	3.4
Total	7061	100.0	6106	100.0
High blood pressure 55/46				
No	5593	79.2	5530	90.6
Yes	1467	20.8	576	9.4
Total	7060	100.0	6106	100.0
Self-report COVID-19				
No, not had COVID-19	6063	89.5	4815	84.4
Yes, have had COVID-19	711	10.5	892	15.6
Total	6774	100.0	5707	100.0
Antibody confirmed SARS-CoV-2				
Negative	2656	91.6	2078	88.8
Positive	244	8.4	261	11.2
Total	2900	100.0	2339	100.0
Hospital admission				
No	2309	98.1	2677	98.1
Yes	44	1.9	52	1.9
Total	2353	100.0	2729	100.0
NHS 111 or 24 h contact				
No	2205	94.03	2538	93.3
Yes	140	5.97	182	6.7
Total	2345	100.0	2720	100.0
Long COVID				
Acute Covid (0–4 weeks)	567	81.4	746	85.2
Long Covid (4 + weeks)	130	18.7	130	14.8
Total	697	100.0	876	100.0

Distribution of key variables among those who responded to at least one COVID-19 survey wave (NCDS *n* = 7769; BCS70 *n* = 7168). Cognitive ability was measured at age 11 in NCDS and age 10 in BCS70, whilst housing tenure was measured at age 7 in NCDS and age 5 in BCS70. For NCDS, potential mediating variables were recorded at age 55, whilst in BCS70 the same variables were recorded at age 46

*SE* standard error

^A^For housing tenure, the additional group “Being Bought” was included in BCS70 but not NCDS

^B^The scale used for self-rated health differed between NCDS and BCS70, so that in NCDS participants rated their health on a scale of “Excellent” to “Poor”, whilst in BCS70 participants were asked to say how true the statement “my health is excellent” was on a scale of “Definitely True” to “Definitely False”

**Fig. 1:**
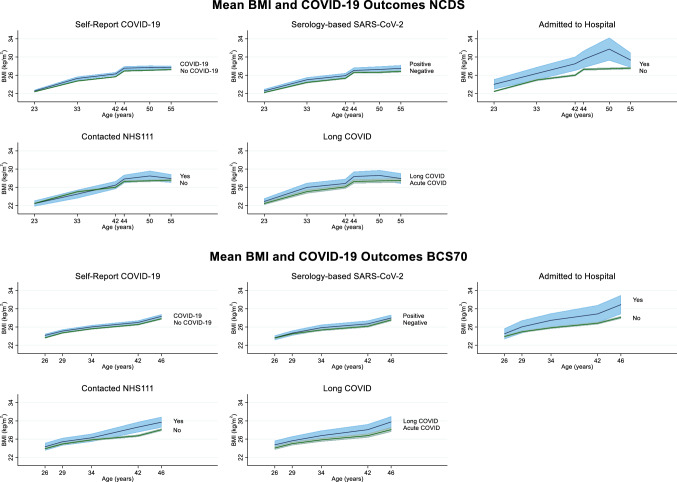
Mean BMI across adulthood by COVID-19 outcomes in NCDS and BCS70. Mean BMI and standard error (SE) across adulthood by COVID-19 outcomes age 62 in NCDS and age 50 in BCS70

Figure 1 shows mean BMI across adulthood by COVID-19 outcome. Mean BMI was typically higher across adulthood for individuals who reported having COVID-19 outcomes, particularly for those who reported a COVID-19 hospital admission.

Figure S2 (Supplementary Material) shows the mean BMI across adulthood by age of first overweight categories and stratified by sex. Typically, those who were first overweight at the youngest observed age had the highest mean BMI at all subsequent ages, whilst those who were never overweight had the lowest mean BMI.

### Regression Results

**Fig. 2:**
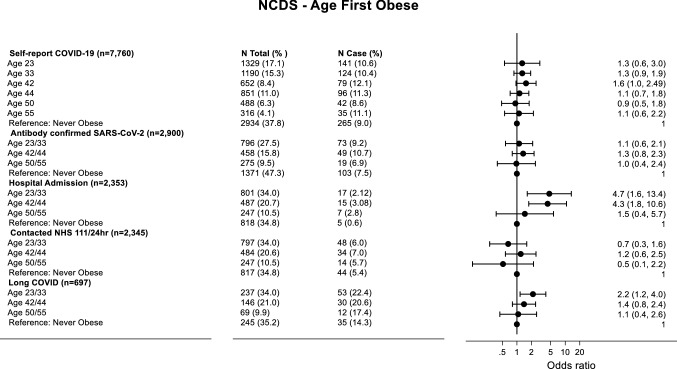
Forrest plot of adjusted logistic regression coefficients for age first obese and COVID-19 outcomes in NCDS. Analysis is adjusted for sex, social class at birth, housing tenure age 7 and cognitive ability age 11. For self-reported COVID-19 and antibody-confirmed SARS-CoV-2 infection the reference category is “no COVID-19” and “no infection”, respectively. For hospital admission and contact with health services, the reference category is “no admission” and “no contact”, respectively. For long COVID the reference category is “acute COVID-19”. *N* total represents the total number of people in each age category, whilst *N* case represents the number of cases within each age category. Log scale used to report Odd Ratios on X axis

**Fig. 3:**
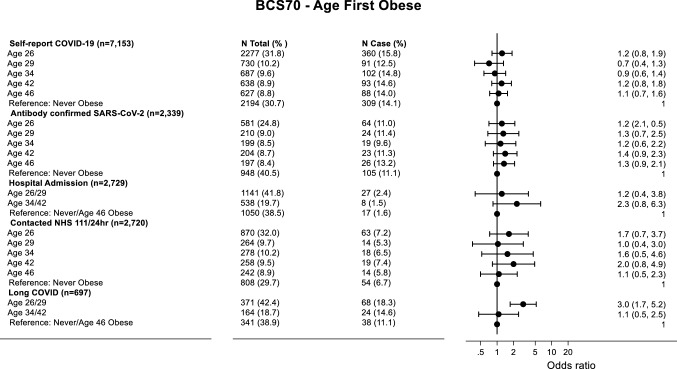
Forrest plot of adjusted logistic regression coefficients for age first obese and COVID-19 outcomes in BCS70. Analysis is adjusted for sex, social class at birth, housing tenure age 5 and cognitive ability age 10. For self-reported COVID-19 and antibody-confirmed SARS-CoV-2 infection the reference category is “no COVID-19” and “no infection”, respectively. For hospital admission and contact with health services, the reference category is “no admission” and “no contact”, respectively. For long COVID the reference category is “acute COVID-19”. *N* total represents the total number of people in each age category, whilst *N* case represents the number of cases within each age category. Log scale used to report Odd Ratios on X axis

Figures 2 and 3 show the associations between the age cohort members were first obese and COVID-19 outcomes in NCDS and BCS70, respectively. In both cohorts, being first obese at a younger age was associated with long COVID, and in NCDS it was also associated with hospital admission and self-reported COVID-19. Cohort members that were first obese age 23/33 in NCDS were over twice as likely to develop long COVID in adjusted models (OR 2.15, 95% CI 1.17–4.00) compared to those never obese. In BCS70 cohort members first obese at age 26/29 were three times as likely to develop long COVID (OR 3.01, 95% CI 1.74–5.22) compared to those never obese/first obese at age 46. In NCDS individuals first obese at ages 23/33 were over four times as likely (OR 4.69, 95% CI 1.64–13.39) to be admitted to the hospital because of COVID-19 compared to those never obese, with a similar association observed for age 42/44. Whilst no association was seen for being first obese at age 26/29 and hospital admission in BCS70, there was a large effect size for the first age of obesity at 34/42 compared to those never obese/first obese at age 46, although confidence intervals were wide (OR 2.30, 95% CI 0.84–6.29). In NCDS first being obese at age 42 (OR 1.55, 95% CI 0.96–2.50) was associated with self-reported COVID-19 compared to those never obese.

In NCDS, earlier age first overweight was only associated with self-reported COVID-19 and serology-based infection with SARS-CoV-2. In adjusted models, cohort members who were first overweight at age 23/33 were more likely to self-report COVID-19 (age 23 OR: 1.54, 95% CI 1.03–2.32; age 33 OR 1.44, 95% CI 1.03–2.01), and cohort member that were first overweight at ages 23/33 were more likely to have serology-confirmed SARS-CoV-2 (OR 1.53, 95% CI 0.93–2.5) compared to those never overweight (Figure S3 (Supplementary Material)). In BCS70, earlier age first overweight was only associated with contacting NHS111. Cohort members who were first overweight at age 26 were more likely (OR 1.83, 95% CI 0.96–3.45) to contact NHS 111 regarding their COVID-19 symptoms compared to those never overweight (Figure S4 (Supplementary Material)).

The sensitivity analysis using self-reported COVID-19 at wave 3 only (Table S2) was largely similar to the main analysis, but with a slightly larger odds ratio for first becoming overweight at 23 and self-reported COVID-19 in NCDS. The sensitivity analysis without COVID-19 and IP weights differed slightly from the main analysis, especially for results regarding the age of first overweight, whilst results for age first obese were more consistent with the main analysis. Further details are given in Table S3 and S4 and results S1 (Supplementary Material).

#### Secondary Analysis: Mediation

Table 2 shows the odds ratios for COVID-19 outcomes per 5 kg/m^2^ greater BMI, controlling for confounders, and then sequentially adjusted for additional potential mediators. This allowed estimation of the total effect (model 1), followed by estimation of the direct effect, not going through respective mediators, in the subsequent models (models 2–7). The BMI observation ages included in the mediation analysis were dependent on a significant association (*p* < 0.1) being identified for first becoming overweight or obese at the same age in the primary analysis.

**Table 2:** Change in odds of COVID-19 outcomes per 5 kg/m^2^ in BMI in NCDS and BCS70 adjusting for potential mediators

	Odds ratio	Lower CI	Upper CI	*P* value	Odds Ratio	Lower CI	Upper CI	*P* value
1958 National child development study (NCDS)								
Self-Reported COVID-19	BMI age 23				BMI age 33			
M1: sex, social class, cognitive ability, tenure	1.15	0.97	1.36	0.11	1.13	1.01	1.26	0.043
M2: M1 + BMI age 55	–	–	–	–	1.11	0.95	1.31	0.18
M3: M1 + Diabetes Age 55	–	–	–	–	1.10	0.98	1.23	0.11
M4: M1 + blood pressure age 55	–	–	–	–	1.12	1.00	1.26	0.063
M5: M1 + self-rated health age 55	–	–	–	–	1.13	1.00	1.26	0.047
M6: M1 + diabetes age 55, blood pressure age 55	–	–	–	–	1.09	0.98	1.23	0.13
M7: M1 + all mediators	–	–	–	–	1.10	0.93	1.29	0.26
Self-Reported COVID-19	BMI age 42							
M1: sex, social class, cognitive ability, tenure	1.15	1.02	1.29	0.022				
M2: M1 + BMI age 55	1.19	1.00	1.43	0.054				
M3: M1 + diabetes age 55	1.12	1.00	1.26	0.057				
M4: M1 + blood pressure age 55	1.14	1.01	1.29	0.032				
M5: M1 + self-rated health age 55	1.15	1.02	1.29	0.022				
M6: M1 + diabetes age 55, blood pressure age 55	1.11	0.99	1.26	0.07				
M7: M1 + All Mediators	1.17	0.98	1.40	0.088				
Antibody confirmed SARS-CoV-2	BMI age 23				BMI age 33			
M1: sex, social class, cognitive ability, tenure	1.27	0.99	1.65	0.066	1.16	0.97	1.41	0.11
M2: M1 + BMI age 55	1.29	0.91	1.81	0.15	–	–	–	–
M3: M1 + diabetes age 55	1.23	0.93	1.61	0.14	–	–	–	–
M4: M1 + blood pressure age 55	1.26	0.98	1.64	0.078	–	–	–	–
M5: M1 + self-rated health age 55	1.29	1.00	1.67	0.056	–	–	–	–
M6: M1 + diabetes age 55, blood pressure age 55	1.22	0.93	1.60	0.15	–	–	–	–
M7: M1 + all mediators	1.23	0.86	1.76	0.25	–	–	–	–
Hospital admission	BMI age 23				BMI age 33			
M1: sex, social class, cognitive ability, tenure	2.06	1.42	2.97	< 0.001	1.38	1.08	1.75	0.009
M2: M1 + BMI age 55	1.78	1.05	3.02	0.033	1.13	0.85	1.50	0.40
M3: M1 + diabetes age 55	1.84	1.20	2.83	0.005	1.29	1.00	1.66	0.052
M4: M1 + blood pressure age 55	1.88	1.28	2.75	0.001	1.32	1.04	1.68	0.025
M5: M1 + self rated health age 55	1.93	1.33	2.77	< 0.001	1.33	1.05	1.70	0.02
M6: M1 + diabetes age 55, blood pressure age 55	1.73	1.11	2.69	0.015	1.25	0.96	1.63	0.10
M7: M1 + All Mediators	1.69	0.93	3.10	0.086	1.12	0.80	1.55	0.51
Hospital Admission	BMI age 42				BMI age 44			
M1: sex, social class, cognitive ability, tenure	1.57	1.18	2.08	0.002	1.60	1.22	2.10	0.001
M2: M1 + BMI age 55	1.36	0.88	2.11	0.17	1.64	0.89	3.02	0.12
M3: M1 + diabetes age 55	1.44	1.03	2.00	0.035	1.49	1.09	2.02	0.012
M4: M1 + blood pressure age 55	1.47	1.09	1.98	0.011	1.51	1.15	1.99	0.003
M5: M1 + self-rated health age 55	1.46	1.10	1.95	0.009	1.53	1.17	2.00	0.002
M6: M1 + diabetes age 55, blood pressure age 55	1.37	0.97	1.93	0.075	1.43	1.04	1.96	0.027
M7: M1 + all mediators	1.25	0.75	2.08	0.38	1.63	0.82	3.26	0.17
Long COVID	BMI age 23				BMI age 33			
M1: sex, social class, cognitive ability, tenure	1.29	0.89	1.84	0.17	1.19	0.96	1.48	0.11
M2: M1 + BMI age 55	–	–	–	–	–	–	–	–
M3: M1 + diabetes age 55	–	–	–	–	–	–	–	–
M4: M1 + blood pressure age 55	–	–	–	–	–	–	–	–
M5: M1 + self-rated health age 55	–	–	–	–	–	–	–	–
M6: M1 + diabetes age 55, blood pressure age 55	–	–	–	–	–	–	–	–
M7: M1 + all mediators	–	–	–	–	–	–	–	–
1970 British cohort study (BCS70)								
NHS 111	BMI age 26							
M1:, sex, social class, cognitive ability, tenure	1.2	0.93	1.55	0.16				
M2: M1 + BMI age 46	–	–	–	–				
M3: M1 + diabetes age 46	–	–	–	–				
M4: M1 + blood pressure age 46	–	–	–	–				
M5: M3 + blood pressure age 46	–	–	–	–				
M6: M1 + self-rated health age 46	–	–	–	–				
M7: M1 + all mediators	–	–	–	–				
Long COVID	BMI age 26				BMI age 29			
M1:, sex, social class, cognitive ability, tenure	1.40	1.07	1.83	0.015	1.28	1.01	1.62	0.04
M2: M1 + BMI age 55	1.18	0.77	1.79	0.46	1.01	0.67	1.52	0.97
M3: M1 + diabetes age 55	1.32	0.99	1.77	0.063	1.22	0.96	1.55	0.11
M4: M1 + blood pressure age 55	1.35	1.03	1.76	0.028	1.23	0.98	1.57	0.081
M5: M1 + self-rated health age 55	1.28	0.96	1.72	0.09	1.19	0.93	1.51	0.17
M6: M1 + diabetes age 55, blood pressure age 55	1.30	0.98	1.74	0.073	1.20	0.94	1.53	0.14
M7: M1 + all mediators	1.07	0.68	1.69	0.78	0.93	0.61	1.42	0.75

Odd ratios represent a change in odds of the outcome per 5 kg/m^2^ increase in BMI. Only those ages where an association was observed (*p* < 0.10) in the main analysis were included in further exploratory mediation analysis. Similarly, mediators were only tested where an association was observed with BMI as a continuous variable when adjusting for sex and social class at birth (*p* < 0.10)

Mediation analysis was also not explored if there was no association between continuous BMI and COVID-19 outcomes, when adjusting for sex, social class at birth, childhood housing tenure and cognitive ability. This was the case for BMI age 23 and self-reported COVID-19, BMI age 23 and 33 with long COVID and BMI age 33 with serology-confirmed SARS-CoV-2 infection in NCDS, and BMI at age 26 and contacting NHS 111 in BCS70.

For the remaining associations, a 5 kg/m^2^ increase in BMI was typically associated with 13–40% greater odds of the respective COVID-19 outcome, representing the total effect. However, in NCDS, a 5 kg/m^2^ increase in BMI at age 23 was associated with twice the odds of admission to hospital with COVID-19 (OR 2.06, 95% CI 1.42–2.97), and a 5 kg/m^2^ increase in BMI at 33, 42 and 44 was associated with 38–60% greater odds of hospital admission.

In NCDS, adding contemporaneous BMI as a mediator partially attenuated associations for the majority of outcomes (Model 2), as did including both blood pressure and diabetes (Model 6). However, for some associations there was evidence of collinearity between historic and contemporaneous BMI. In BCS70, all the associations were attenuated by contemporaneous BMI (Model 2). In both cohorts, the inclusion of all mediators (Model 7) resulted in limited or no evidence of an association between BMI and most COVID-19 outcomes.

The exceptions to this were for BMI at 23 and hospital admission and BMI at 42 and self-reported COVID-19 in NCDS. These associations were not significantly attenuated with the inclusion of additional mediators (Models 2–5). Once all mediators were included (Model 7), a 5 kg/m^2^ increase in BMI at age 23 remained associated with 69% greater odds of admission to hospital with COVID-19 (OR 1.69, 95% CI 0.93–3.10, *p* = 0.086), representing the direct effect not going through mediators. Similarly, and a 5 kg/m^2^ higher BMI at age 42 was associated with 17% greater odds of self-reported COVID-19 (OR 1.17, 95% CI 0.98–1.40, *p* = 0.088).

## Discussion

In two nationally representative British birth cohorts, we estimated the risk of severe COVID-19 outcomes, including long COVID, associated with the age at which participants first become overweight or obese, compared to those who are never overweight or obese, respectively. We found that an earlier age of first obesity was related to long COVID and COVID-19 severity, although results were mixed and, in some analyses, underpowered due to the relatively small number of individuals experiencing the outcome. In both cohorts, there was consistent evidence that becoming obese earlier in adulthood was associated with an increased likelihood of long COVID. There was greater evidence in NCDS—the earlier born cohort—that becoming obese in early adulthood was associated with COVID-19 and COVID-19 severity: there was over four times the risk of hospital admission for those first obese at 23 or 33 relative to those never obese. Although there was less evidence in the later born, and therefore younger, cohort that becoming overweight or obese in early adulthood was related to COVID-19 and COVID-19 severity, there was some evidence that becoming overweight at a younger age was associated with contacting NHS 111. Most associations with BMI across early adulthood could be partially explained by BMI in later life, or a combination of diabetes and high blood pressure. However, BMI in earlier life remained associated with hospital admission and self-reported COVID-19 even once accounting for potential mediators.

Previous research has found an earlier age of overweight and obesity is associated with chronic kidney disease, cardiovascular disease, high blood pressure, cholesterol and diabetes [18–21]. To the best of our knowledge, no previous research has looked at associations between the timing of first overweight and obesity and COVID-19 outcomes. Overweight and obesity have previously been found to be associated with COVID-19 severity [15] and long COVID [5, 16, 17]. For hospital admission, ICU admission and deaths, a J-shape curve has previously been observed where those at both the lowest and highest end of the BMI distribution have an increased risk of high-severity COVID-19 [41]. A linear association was observed for BMI higher than 23 kg/m^2^, and the effect of higher BMI on severity was particularly notable for those under the age of 40. Similarly, we observe associations for earlier age of obesity and hospital admissions; however, we find stronger associations between early-life BMI and hospital admission among older adults as opposed to younger. We were unable to test associations with ICU admission or deaths as this information was not available.

Previous research among 10 longitudinal studies in the UK, found overweight and obesity were associated with an increased likelihood of long COVID [5], whilst long COVID was more likely with increasing BMI among individuals participating in the COVID symptoms study [17]. Our results additionally demonstrate a consistent association between being obese, but not overweight, in earlier adulthood and long COVID in two cohorts. It is possible that inflammation as a result of obesity in earlier life results in long-term changes to the immune system that have consequences for COVID-19 severity and long COVID in later adult life. A systematic review of 26 reviews found childhood obesity may reduce immune responsiveness to vaccines and microorganisms [12]. Pathways between obesity, impaired immune function and COVID-19 have been highlighted in a review that emphasises the role of low-grade inflammation on reducing the body’s immune system’s ability to respond rapidly to infections [42].

Similar to previous research in UK Biobank which has looked at BMI and contracting the SARS-CoV-2 virus [13, 14], we find that in the NCDS a younger age of overweight and obesity is related to higher self-reported COVID-19 and serology-based SARS-CoV-2 infection rates. It is possible that this association is explained by differences in exposure to the virus, that differ for individuals with higher BMI. Socioeconomic position (SEP) is associated with BMI, and also with the likelihood of living in overcrowded homes or working in occupations that cannot accommodate working from home, thus increasing the risk of COVID-19 exposure [43].

In secondary analyses, we investigated the possible mediating roles of diabetes, high blood pressure, and BMI in later adult life. Although we found the combined effect of high blood pressure and diabetes to have some attenuating effect for some of the observed associations in NCDS, we found BMI in later adult life to have the most consistent and substantial attenuating effects for the considered outcomes in both cohorts. This is likely reflective of that those who become overweight earlier having a higher final BMI, which largely mediates the associations for some outcomes. By including both BMI in early life and BMI in later life in mediating models, we tried to examine whether the timing of first overweight or obesity was important for COVID-19 outcomes independently of BMI in later life. However, this study assumed that for most individuals once they had become overweight or obese, they were likely to stay in that weight category or increase their weight category. Due to small numbers, we were unable to test if reductions in BMI later in the life course would have changed the association with COVID-19 outcomes in later life.

### Strengths and Limitations

This study was conducted in two nationally representative cohorts in Britain that have collected BMI across adult life, allowing for exploration of how life course experience of overweight and obesity is related to COVID-19, COVID-19 severity and long COVID. We estimated the risk of severe COVID-19 outcomes associated with age at first obesity compared to those who never became obese, and, by comparing results in two cohorts, born 12 years apart, we were able to explore differences between cohorts and ages. This study utilised multiple imputations to address missing data in the exposures, covariates and mediators meaning bias attributable to missing data was reduced. However, due to small numbers of individuals experiencing certain outcomes (i.e. hospital admission, long COVID), it was still necessary to collapse the age of the first overweight and obesity groups for these analyses, resulting in a potential loss of information. Nonetheless, analysis was still underpowered for certain outcomes such as hospital admission and long COVID due to the small study sample with each outcome. A pooled analysis of the cohorts was not run due to the different way the exposure—the age cohort members were first overweight or obese—was operationalised, as the ages of BMI measures did not correspond between the two cohorts.

Another advantage of this study is that serological data on SARS-CoV-2 infection were available in addition to self-reported COVID-19. Therefore, analysis was not wholly reliant on self-report, which is prone to misreporting, especially early in the pandemic where testing was not widely available. However, the remaining variables included were reliant on self-report, and this may have introduced misclassification that may have either upwardly or downwardly biased results dependent on if COVID-19 outcomes were over on underreported.

Analysis was conducted on cohort members who responded to wave 1–3 for self-reported COVID-19. However, combining across sweeps may have resulted in individuals who had not responded to the final sweep being misclassified as not having had COVID-19 if they had then gone on to be infected. To address this, sensitivity analysis explored associations for only those individuals that responded at wave 3. However, as numbers reporting accessing health care services because of COVID-19 were low, this was not possible for hospital admission or contact with NHS 111. It is therefore possible that individuals who responded negatively in earlier sweeps but did not respond to the final sweep were misclassified as not having accessed healthcare services when they in fact had.

Individuals reporting COVID-19 the same month they completed the survey were removed from the long COVID analysis, to prevent misclassification. However, individuals reporting a COVID-19 illness in the month prior to their response to the survey may have had COVID-19 less than 4 weeks prior. It is therefore possible that these individuals had not had enough time for long COVID to develop. It was not possible to identify these individuals as the specific day in each month of COVID-19 illness was not reported, but given the COVID-19 rates the numbers potentially affected are likely to be low.

COVID-19 and IP weights were used to address selection bias and index event bias, and unweighted results were presented in a sensitivity analysis for comparison. Unweighted analyses demonstrated slightly different findings to the main results, especially for the earliest age of overweight, indicating that analysis is relatively sensitive to the approach employed. This work highlights a need for researchers to utilise appropriate methods when conducting longitudinal analysis that experiences attrition, and to consider possible index event bias for research where the outcome is long COVID.

A number of early life variables were considered in the analysis to address potential confounding. However, because the exposure—the age at which cohort members were first overweight or obese—covered adult life, confounders in adulthood were not adjusted for, as they would not causally precede the exposure, and may instead represent mediators. Therefore, it is possible that there were unmeasured or residual confounders that were not accounted for. Secondary analysis did explore possible mediators, by including them in regression analysis with BMI as a continuous measure, at the ages obesity or overweight onset was found to be significant in the primary analysis. However, the mediation analysis did not adopt formal causal mediation methods, and in particular, did not account for possible interactions between the exposure and mediator. Future research could adopt more formal methods to assess mediation.

## Conclusions

In two nationally representative British birth cohorts, the age of first becoming overweight and obese was not related to COVID-19 and COVID-19 severity in a consistent way, although in both cohorts there was consistent evidence of increased risk of long COVID for individuals who first experienced obesity at a younger age, compared to those who were never obese. We found greater evidence of an association between early life overweight and obesity and COVID-19 outcomes, particularly for hospital admission, in NCDS, the earlier born and therefore older aged cohort. That an earlier age of becoming overweight or obese is associated with COVID-19 outcomes highlights the potential life course effect of obesity as a state of low-grade inflammation, and the consequences this may have on the immune system.

## Supplementary Information

Below is the link to the electronic supplementary material.

Supplementary file1 (DOCX 182 KB)

## Data Availability

Data for NCDS (SN 6137) and BCS70 (SN 8547) and all four COVID-19 surveys (SN 8658) are available through the UK Data Service.

## Abbreviations

BCS70: 1970 British cohort study
BMI: Body mass index
COVID-19: Coronavirus disease
ICU: Intensive care units
NICE: National institute for health and care excellence
NCDS: 1958 National child development study
NHS: National health service
SEP: Socioeconomic position
UK: United Kingdom
