# Supplementary material for: Age of First Overweight and Obesity, COVID-19 and Long COVID in Two British Birth Cohorts
Source: J Epidemiol Glob Health. 2023 Feb 22;13(1):140–53. doi: 10.1007/s44197-023-00093-5 (PMC9945825; doi:10.1007/s44197-023-00093-5)
Supplement: Supplementary file 1 — Supplementary file1 (DOCX 182 KB) [file 44197_2023_93_MOESM1_ESM.docx]

Supplementary Material

# Figure S1. Directed acylic graph showing exporure, outcome and confounders


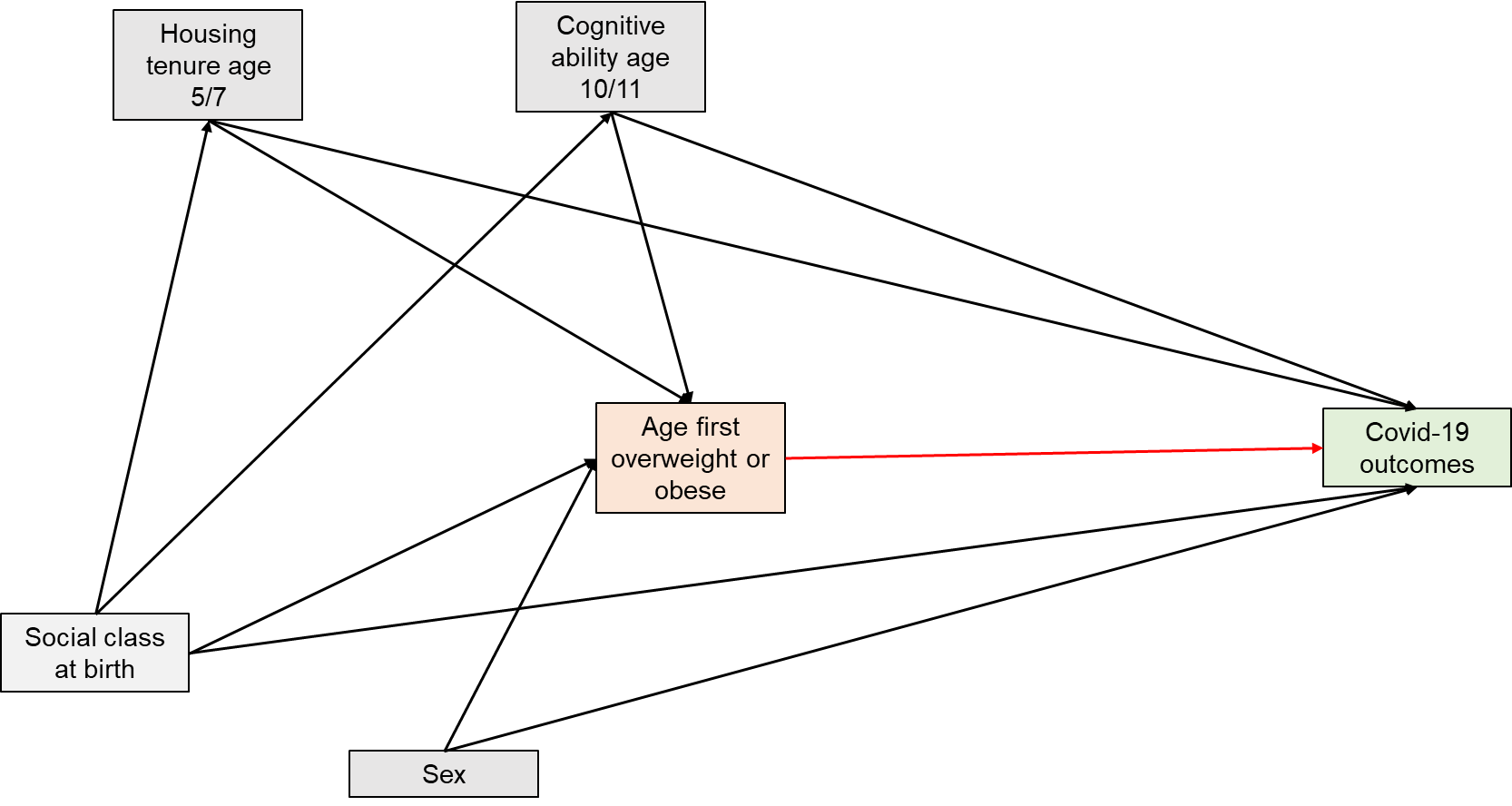


**Figure S1.** Directed acyclic graph showing exposure (age first overweight and obese), outcome (COVID-19 outcomes) and confounders (sex, social class at birth, housing tenure age 5/7 and cognitive ability age 10/11) in the primary analysis. The red path indicates the causal path, whilst black paths indicate biasing paths that are removed when controlling for the confounders.

# Table S1. Exploration of Missing Data

| Variable | Missing (N) | % |
| --- | --- | --- |
| **NCDS (N = 7,769)** |  | |
| Lifetime Height | 56 | 0.72 |
| Weight age 23 | 1,191 | 15.33 |
| Weight age 33 | 1,143 | 14.71 |
| Weight age 42 | 804 | 10.35 |
| Weight age 44* | 1,364 | 17.56 |
| Weight at 50 | 1,116 | 14.36 |
| Weight at 55 | 1,095 | 14.09 |
| Sex | 0 | 0.00 |
| Social Class at Birth | 401 | 5.16 |
| Housing Tenure age 7 | 966 | 12.43 |
| Cognitive Ability age 11 | 1,042 | 13.41 |
| Self-Rated Health at 55 | 698 | 8.98 |
| Blood Pressure at 55 | 709 | 9.13 |
| Diabetes at 55 | 708 | 9.11 |
| **BCS70 (N = 7,168)** |  | |
| Lifetime Height | 120 | 1.67 |
| Weight age 26 | 1,924 | 26.8 |
| Weight age 29 | 1,101 | 15.4 |
| Weight age 34 | 1,524 | 21.3 |
| Weight age 42 | 1,373 | 19.2 |
| Weight age 46* | 1,672 | 23.3 |
| Sex | 5 | 0.07 |
| Social Class at Birth | 529 | 7.38 |
| Housing Tenure age 5 | 1,286 | 17.94 |
| Cognitive Ability age 10 | 1,960 | 27.24 |
| Self-Rated Health at 46 | 1,334 | 18.61 |
| Blood Pressure at 46 | 1,062 | 14.82 |
| Diabetes at 46 | 1,062 | 14.82 |

**Table S1 Footnote:** *Nurse Measured. Percentage of the sample missing for each variable is calculated for those individuals that responded to at least one wave of the COVID-19 surveys (NCDS N=7,769; BCS70 N=7,168). In NCDS, 42% of those individuals that responded to at least one of the COVID-19 surveys had complete data on all predictors, covariates and mediators, whilst 29% had complete data in BCS70. Lifetime height and sex had the lowest level of missing data in both cohorts (<2.0%) followed by social class at birth (5.2-7.4%). In NCDS the variables with the highest level of missing data were weight at age 44 (17.6%) and at age 23 (15.3%). In BCS70 the most missing data was found for cognitive ability at age 10 (27.2%), weight at age 26 (26.8%) and age 46 (23.3%).

# Methods S1. Additional Information for Imputation Models

For some outcomes, social class at birth in BCS70 was collapsed to fewer categories or groups were recoded to missing due to low counts preventing convergence of imputation models. BMI at each age across adulthood was derived as a passive variable from the imputed lifetime height and imputed weight across adulthood. From this, age of first overweight and obesity were also derived as passive variables, using the methods described in the main text. Imputation models were fitted as described in the statistical analysis within each imputed dataset and combined using Rubin’s rules.

Auxiliary variables were selected for their anticipated associations with the underlying values of analysis variables subject to missingness or their association with the probability of non-response based on previous work [30].

**Auxiliary variables in NCDS:** highest academic qualification up to 46, cholesterol at 44, self-rated health at 42, disability at 46, weight at 16, height at 16, dad’s height (reported when cohort members were age 7), mum’s height (cohort members age 7), dad’s height (cohort members age 7) and mum’s height (cohort members age 7).

**Auxiliary variables in BCS70:** highest academic qualification obtained up to 42, disability at 42, self-rated health at 38, total Cholesterol at 46, nurse measured height at 46, weight age 16, height age 16, mum’s weight (reported when cohort members age 5), dad’s weight (cohort members age 5), mum’s height (cohort members age 5), dad’s height (cohort members age 5).

# Methods S2. Additional information on survey weights and inverse probability weights

The present analysis rescaled and truncated the COVID-19 weights to 20 to prevent extreme weights exerting undue influence. For COVID-19 status, only the COVID-19 weight for wave 3 was used. For serology, hospital admission and contact with NHS 111, the mean of the rescaled COVID-19 weights across COVID-19 sweeps were used, truncated to 20.

The product of the IP weights and rescaled COVID-19 weight at wave 3 was used and truncated for values exceeding 20. Variables used to derive IP weights are described below. Multiple imputation was used to address missingness to ensure that it was possible to include all cohort members in the weight-derivation process.

All variables used to derive IPW weights were taken from wave 3 of the COVID-19 surveys, unless otherwise specified:

**Variables used in NCDS:** sex, mental health score, BMI, weekly income, social class at birth, asthma, smoking status, if children live in the household and number of children.

**Variables used in BCS70:** sex, mental health score, BMI, weekly income, social class at birth, asthma, education status (age 42), smoking status, if children live in the household and number of children.

# Figure S2. Mean BMI across adulthood by sex and age first overweight in NCDS and BCS70

**Figure S2.** Mean BMI across adulthood stratified by the age cohort members were first overweight and by sex in NCDS and BCDS70.

# Figure S3. Forrest plot of adjusted logistic regression coefficients for age first overweight and COVID-19 outcomes in NCDS

**Figure S3.** Analysis is adjusted for sex, social class at birth, housing tenure age 7 and cognitive ability age 11. For self-reported COVID-19 and antibody confirmed SARS-CoV-2 infection the reference category is “no COVID-19” and “no infection”, respectively. For hospital admission and contact with health services, the reference category is “no admission” and “no contact”, respectively. For long COVID the reference category is “acute COVID-19”. N total represents the total number of people in each age category, whilst N case represents the number of cases within each age category. Log scale used to report Odd Ratios on X axis.

# Figure S4. Forrest plot of adjusted logistic regression coefficients for age first overweight and COVID-19 outcomes in BCS70

**Figure S4:** Analysis is adjusted for sex, social class at birth, housing tenure age 5 and cognitive ability age 10. For self-reported COVID-19 and antibody confirmed SARS-CoV-2 infection the reference category is “no COVID-19” and “no infection”, respectively. For hospital admission and contact with health services, the reference category is “no admission” and “no contact”, respectively. For long COVID the reference category is “acute COVID-19”. N total represents the total number of people in each age category, whilst N case represents the number of cases within each age category. Log scale used to report Odd Ratios on X axis.

# Table S2. Sensitivity analysis: Self-report COVID-19 measured at wave 3 only

|  | **Age First Overweight** | | | | | | **Age First Obese** | | | | | |
| --- | --- | --- | --- | --- | --- | --- | --- | --- | --- | --- | --- | --- |
| Self-report COVID-19 | N Total (%) | N Case (%) | Odds Ratio | Lower CI | Upper CI | P Value | N Total (%) | N Case (%) | Odds Ratio | Lower CI | Upper CI | P Value |
| **1958 NATIONAL CHILD DEVELOPMENT STUDY (NCDS)** | | | | | | | | | | | | |
| Reference: Never Overweight/Obese | 1,017 (15.0) | 84 (8.3) | 1.00 | - | - | - | 2664 (39.9) | 249 (9.4) | 1.00 | - | - | - |
| Overweight/obese age 23 | 1,872 (27.6) | 216 (11.5) | 1.87 | 1.20 | 2.91 | 0.006 | 1115 (16.5) | 131 (11.8) | 1.79 | 0.67 | 4.79 | 0.24 |
| Overweight/obese age 33 | 1,890 (27.9) | 225 (11.9) | 1.41 | 0.94 | 2.10 | 0.095 | 1023 (15.1) | 112 (11.0) | 1.42 | 0.79 | 2.54 | 0.24 |
| Overweight/obese age 42 | 840 (12.4) | 83 (9.9) | 1.32 | 0.78 | 2.23 | 0.31 | 553 (8.2) | 69 (12.5) | 1.78 | 1.04 | 3.05 | 0.04 |
| Overweight/obese age 44 | 709 (10.5) | 62 (8.7) | 1.07 | 0.63 | 1.81 | 0.82 | 726 (10.7) | 80 (11.0) | 1.14 | 0.65 | 2.00 | 0.64 |
| Overweight/obese age 50 | 262 (3.9) | 27 (10.3) | 1.35 | 0.61 | 2.94 | 0.46 | 423 (6.2) | 40 (9.5) | 0.94 | 0.44 | 2.00 | 0.88 |
| Overweight/obese age 55 | 184 (2.7) | 14 (7.6) | 0.89 | 0.38 | 2.11 | 0.79 | 270 (4.0) | 30 (11.1) | 1.29 | 0.53 | 3.12 | 0.58 |
| **1970**  **BRITISH COHORT STUDY (BCS70)** | | | | | | | | | | | | |
| Reference: Never Overweight/Obese | 797 (14.0) | 109 (13.7) | 1 | - | - | - | 1,869 (32.8) | 282 (15.1) | 1.00 | - | - | - |
| Overweight/obese age 26 | 2,679 (46.9) | 441 (16.5) | 1.07 | 0.74 | 1.55 | 0.72 | 1,725 (30.2) | 298 (17.3) | 1.20 | 0.72 | 1.98 | 0.48 |
| Overweight/obese age 29 | 837 (14.7) | 139 (16.6) | 1.13 | 0.74 | 1.74 | 0.58 | 579 (10.2) | 81 (14.0) | 0.79 | 0.40 | 1.54 | 0.49 |
| Overweight/obese age 34 | 566 (9.9) | 87 (15.4) | 1.16 | 0.71 | 1.91 | 0.55 | 542 (9.5) | 87 (16.1) | 0.94 | 0.53 | 1.66 | 0.83 |
| Overweight/obese age 42 | 450 (7.9) | 62 (13.8) | 0.90 | 0.54 | 1.49 | 0.68 | 501 (8.8) | 79 (15.8) | 1.24 | 0.73 | 2.11 | 0.43 |
| Overweight/obese age 46 | 378 (6.6) | 54 (14.3) | 1.10 | 0.67 | 1.81 | 0.70 | 491 (8.6) | 65 (13.2) | 1.10 | 0.66 | 1.83 | 0.73 |

**Table S2. Footnote :** Multiply imputed logistic regression results for associations between age first obese or overweight and self-reported COVID-19 in NCDS and BCS70, only using self-reported COVID-19 measured at wave 3. Analysis is adjusted for sex, social class at birth, housing tenure age 7 and cognitive ability age 11.

# Table S3. Sensitivity analysis: Unweighted Regression for age first Obese

|  | Odds Ratio | Lower CI | Upper CI | P Value | Odds Ratio | Lower CI | Upper CI | P Value |
| --- | --- | --- | --- | --- | --- | --- | --- | --- |
|  | **Unadjusted** | | | | **Adjusted** | | | |
| **1958 NATIONAL CHILD DEVELOPMENT STUDY (NCDS)** | | | | | | | | |
| **Self-report COVID-19 (n=7760) – reference: no COVID** | | | | | | | | |
| Reference: Never Obese | 1.00 | - | - | - | 1.00 | - | - | - |
| First obese age 23 | 0.95 | 0.55 | 1.66 | 0.87 | 0.90 | 0.52 | 1.59 | 0.73 |
| First obese age 33 | 1.20 | 0.91 | 1.57 | 0.19 | 1.16 | 0.88 | 1.53 | 0.28 |
| First obese age 42 | 1.62 | 1.22 | 2.14 | 0.001 | 1.59 | 1.20 | 2.11 | 0.001 |
| First obese age 44 | 1.25 | 0.95 | 1.64 | 0.11 | 1.22 | 0.93 | 1.60 | 0.15 |
| First obese age 50 | 0.96 | 0.62 | 1.48 | 0.83 | 0.93 | 0.60 | 1.43 | 0.74 |
| First obese age 55 | 1.24 | 0.80 | 1.93 | 0.33 | 1.20 | 0.77 | 1.85 | 0.43 |
| **Antibody confirmed SARS-CoV-2 infection (n=2,900) – Reference: no infection** | | | | | | | | |
| Reference: Never Obese | 1.00 | **-** | **-** | **-** | 1.00 | **-** | **-** | **-** |
| First obese age 23/33 | 1.09 | 0.67 | 1.78 | 0.73 | 1.01 | 0.62 | 1.66 | 0.96 |
| First obese age 42/44 | 1.61 | 1.12 | 2.32 | 0.01 | 1.53 | 1.06 | 2.22 | 0.024 |
| First obese age 50/55 | 0.98 | 0.56 | 1.72 | 0.95 | 0.90 | 0.51 | 1.59 | 0.72 |
| **Hospital Admission (n=2,353) – Reference: no hospital admission** | | | | | | | | |
| Reference: Never Obese | 1.00 | **-** | **-** | **-** | 1.00 | **-** | **-** | **-** |
| First obese age 23/33 | 3.31 | 1.32 | 8.29 | 0.011 | 2.81 | 1.11 | 7.16 | 0.03 |
| First obese age 42/44 | 4.42 | 2.12 | 9.20 | 0.000 | 3.96 | 1.89 | 8.32 | 0.000 |
| First obese age 50/55 | 2.22 | 0.71 | 6.94 | 0.17 | 1.90 | 0.60 | 6.02 | 0.28 |
| **Contacted NHS 111/24hr (n=2,345) – Reference: no NHS contact** | | | | | | | | |
| Reference: Never Obese | 1.00 | **-** | **-** | **-** | 1.00 | **-** | **-** | **-** |
| First obese age 23/33 | 0.97 | 0.53 | 1.77 | 0.93 | 0.88 | 0.47 | 1.61 | 0.67 |
| First obese age 42/44 | 1.50 | 0.97 | 2.31 | 0.071 | 1.36 | 0.87 | 2.12 | 0.17 |
| First obese age 50/55 | 0.70 | 0.30 | 1.61 | 0.40 | 0.66 | 0.29 | 1.54 | 0.34 |
| **Long COVID (n=697) – Reference: acute COVID** | | | | | | | | |
| Reference: Never Obese | 1.00 | **-** | **-** | **-** | 1.00 | **-** | **-** | **-** |
| First obese age 23/33 | 2.10 | 1.19 | 3.70 | 0.011 | 2.16 | 1.17 | 4.00 | 0.014 |
| First obese age 42/44 | 1.47 | 0.88 | 2.44 | 0.14 | 1.37 | 0.81 | 2.34 | 0.24 |
| First obese age 50/55 | 0.98 | 0.44 | 2.22 | 0.97 | 0.95 | 0.42 | 2.18 | 0.91 |
| **1970**  **BRITISH COHORT STUDY (BCS70)** | | | | | | | | |
| **Self-report COVID-19 (n=7153) – reference: no COVID** | | | | | | | | |
| Reference: Never Obese | 1.00 | **-** | **-** | **-** | 1.00 | **-** | **-** | **-** |
| First obese age 26 | 1.40 | 1.07 | 1.82 | 0.013 | 1.40 | 1.07 | 1.82 | 0.013 |
| First obese age 29 | 0.82 | 0.58 | 1.14 | 0.24 | 0.81 | 0.58 | 1.14 | 0.23 |
| First obese age 34 | 1.06 | 0.79 | 1.43 | 0.71 | 1.06 | 0.78 | 1.43 | 0.71 |
| First obese age 42 | 1.28 | 1.00 | 1.65 | 0.053 | 1.30 | 1.01 | 1.67 | 0.045 |
| First obese age 46 | 1.11 | 0.86 | 1.42 | 0.43 | 1.11 | 0.86 | 1.42 | 0.43 |
| **Antibody confirmed SARS-CoV-2 infection (N=** **2,339) – Reference: no infection** | | | | | | | | |
| Reference: Never Obese | 1.00 | **-** | **-** | **-** | 1.00 | **-** | **-** | **-** |
| First obese age 26 | 1.25 | 0.72 | 2.14 | 0.43 | 1.22 | 0.71 | 2.12 | 0.47 |
| First obese age 29 | 1.23 | 0.65 | 2.34 | 0.53 | 1.30 | 0.68 | 2.50 | 0.43 |
| First obese age 34 | 1.19 | 0.63 | 2.24 | 0.60 | 1.16 | 0.61 | 2.21 | 0.66 |
| First obese age 42 | 1.43 | 0.88 | 2.34 | 0.15 | 1.41 | 0.86 | 2.32 | 0.18 |
| First obese age 46 | 1.38 | 0.89 | 2.13 | 0.15 | 1.34 | 0.86 | 2.09 | 0.20 |
| **Hospital Admission (n=2,729) – Reference: no hospital admission** | | | | | | | | |
| Reference: Never Obese/ Obese age 46 | 1.00 | **-** | **-** | **-** | 1.00 | **-** | **-** | **-** |
| First obese 26/29 | 1.70 | 0.79 | 3.69 | 0.18 | 1.58 | 0.72 | 3.46 | 0.25 |
| First obese 34/42 | 1.74 | 0.79 | 3.80 | 0.17 | 1.62 | 0.73 | 3.58 | 0.23 |
| **Contacted NHS 111/24hr (n=2,720) – Reference: no NHS contact** | | | | | | | | |
| Reference: Never Obese | 1.00 | **-** | **-** | **-** | 1.00 | **-** | **-** | **-** |
| First obese age 26 | 1.70 | 1.01 | 2.89 | 0.048 | 1.61 | 0.94 | 2.76 | 0.082 |
| First obese age 29 | 1.11 | 0.51 | 2.39 | 0.80 | 1.14 | 0.53 | 2.47 | 0.74 |
| First obese age 34 | 1.17 | 0.59 | 2.31 | 0.66 | 1.16 | 0.58 | 2.31 | 0.68 |
| First obese age 42 | 1.83 | 1.08 | 3.09 | 0.024 | 1.79 | 1.06 | 3.04 | 0.031 |
| First obese age 46 | 1.12 | 0.61 | 2.05 | 0.72 | 1.08 | 0.58 | 1.99 | 0.82 |
| **Long COVID (n= 876) – Reference: acute COVID** | | | | | | | | |
| Reference: Never Obese/ Obese age 46 | 1.00 | **-** | **-** | **-** | 1.00 | **-** | **-** | **-** |
| First obese 26/29 | 2.52 | 1.55 | 4.09 | 0.000 | 2.59 | 1.57 | 4.30 | 0.000 |
| First obese 34/42 | 1.05 | 0.54 | 2.04 | 0.90 | 1.05 | 0.53 | 2.06 | 0.90 |

**Table S3. Footnote:** Multiply imputed logistic regression results for associations between age first obese and COVID-19 outcomes in NCDS and BCS70 without use of COVID-19 and IP weights. In NCDS, “never obese” is the reference category. In BCS70, “Never obese/First obese 46” is the reference category.

# Table S4. Sensitivity analysis: Unweighted Regression for age first Overweight

|  | Odds Ratio | Lower CI | Upper CI | P Value | Odds Ratio | Lower CI | Upper CI | P Value |
| --- | --- | --- | --- | --- | --- | --- | --- | --- |
|  | **Unadjusted** | | | | **Adjusted** | | | |
| **1958 NATIONAL CHILD DEVELOPMENT STUDY (NCDS)** | | | | | | | | |
| **Self-report COVID-19 (n=7,760) – reference: no COVID** | | | | | | | | |
| Reference: Never Overweight | 1.00 | **-** | **-** | **-** | 1.00 | **-** | **-** | **-** |
| overweight age 23 | 1.39 | 1.07 | 1.80 | 0.01 | 1.36 | 1.04 | 1.78 | 0.03 |
| overweight age 33 | 1.48 | 1.19 | 1.85 | <0.001 | 1.48 | 1.18 | 1.86 | 0.001 |
| overweight age 42 | 1.30 | 0.99 | 1.70 | 0.06 | 1.31 | 0.99 | 1.72 | 0.06 |
| overweight age 44 | 1.20 | 0.90 | 1.61 | 0.21 | 1.20 | 0.90 | 1.60 | 0.22 |
| overweight age 50 | 1.38 | 0.92 | 2.07 | 0.12 | 1.37 | 0.92 | 2.06 | 0.13 |
| overweight age 55 | 1.17 | 0.70 | 1.96 | 0.55 | 1.15 | 0.68 | 1.93 | 0.61 |
| **Antibody confirmed SARS-CoV-2 infection (n=2,900) – Reference: no infection** | | | | | | | | |
| Reference: Never Overweight | 1.00 | **-** | **-** | **-** | 1.00 | **-** | **-** | **-** |
| overweight age 23/33 | 1.49 | 1.04 | 2.13 | 0.031 | 1.34 | 0.93 | 1.95 | 0.12 |
| overweight age 42/44 | 1.48 | 1.01 | 2.16 | 0.046 | 1.38 | 0.94 | 2.04 | 0.11 |
| overweight age 50/55 | 0.96 | 0.50 | 1.83 | 0.90 | 0.92 | 0.48 | 1.76 | 0.80 |
| **Hospital Admission (n=2,353) – Reference: no hospital admission** | | | | | | | | |
| Reference: Never Overweight | 1.00 | **-** | **-** | **-** | 1.00 | **-** | **-** | **-** |
| overweight age 23/33 | 2.47 | 0.92 | 6.61 | 0.07 | 1.87 | 0.69 | 5.09 | 0.22 |
| overweight age 42/44 | 1.68 | 0.56 | 5.02 | 0.35 | 1.34 | 0.44 | 4.02 | 0.61 |
| overweight age 50/55 | 0.86 | 0.10 | 7.65 | 0.89 | 0.75 | 0.083 | 6.70 | 0.79 |
| **Contacted NHS 111/24hr (n=2,345) – Reference: no NHS contact** | | | | | | | | |
| Reference: Never Overweight | 1.00 | **-** | **-** | **-** | 1.00 | **-** | **-** | **-** |
| overweight age 23/33 | 1.12 | 0.67 | 1.86 | 0.67 | 1.11 | 0.66 | 1.87 | 0.70 |
| overweight age 42/44 | 1.71 | 1.00 | 2.92 | 0.049 | 1.70 | 0.98 | 2.94 | 0.058 |
| overweight age 50/55 | 1.98 | 0.99 | 3.94 | 0.053 | 1.80 | 0.90 | 3.61 | 0.099 |
| **Long COVID (n=697) – Reference: acute COVID** | | | | | | | | |
| Reference: Never Overweight | 1.00 | **-** | **-** | **-** | 1.00 | **-** | **-** | **-** |
| overweight age 23/33 | 1.19 | 0.68 | 2.07 | 0.54 | 1.30 | 0.73 | 2.33 | 0.38 |
| overweight age 42/44 | 0.91 | 0.49 | 1.72 | 0.78 | 0.96 | 0.50 | 1.86 | 0.91 |
| overweight age 50/55 | 1.47 | 0.61 | 3.57 | 0.39 | 1.32 | 0.54 | 3.22 | 0.55 |
| **1970**  **BRITISH COHORT STUDY (BCS70)** | | | | | | | | |
| **Self-report COVID-19 (n=7153) – reference: no COVID** | | | | | | | | |
| Reference category: Never Overweight | 1.00 | **-** | **-** | **-** | 1.00 | **-** | **-** | **-** |
| overweight age 26 | 1.29 | 1.06 | 1.56 | 0.011 | 1.30 | 1.07 | 1.59 | 0.010 |
| overweight age 29 | 1.37 | 1.08 | 1.75 | 0.010 | 1.39 | 1.09 | 1.78 | 0.009 |
| overweight age 34 | 1.29 | 0.98 | 1.68 | 0.067 | 1.30 | 0.99 | 1.71 | 0.06 |
| overweight age 42 | 1.25 | 0.94 | 1.67 | 0.13 | 1.26 | 0.94 | 1.68 | 0.13 |
| overweight age 46 | 1.29 | 0.99 | 1.70 | 0.064 | 1.30 | 0.99 | 1.70 | 0.063 |
| **Antibody confirmed SARS-CoV-2 infection (N=** **2,339) – Reference: no infection** | | | | | | | | |
| Reference: Never Overweight | 1.00 | **-** | **-** | **-** | 1.00 | **-** | **-** | **-** |
| overweight age 26 | 1.14 | 0.80 | 1.63 | 0.46 | 1.21 | 0.84 | 1.75 | 0.31 |
| overweight age 29 | 1.45 | 0.96 | 2.21 | 0.079 | 1.55 | 1.01 | 2.37 | 0.046 |
| overweight age 34 | 0.81 | 0.47 | 1.40 | 0.44 | 0.81 | 0.47 | 1.41 | 0.46 |
| overweight age 42 | 1.03 | 0.61 | 1.73 | 0.91 | 1.04 | 0.61 | 1.75 | 0.90 |
| overweight age 46 | 1.32 | 0.79 | 2.21 | 0.29 | 1.33 | 0.80 | 2.24 | 0.28 |
| **Hospital Admission (n=2,729) – Reference: no hospital admission** | | | | | | | | |
| Reference: Never Overweight/ Overweight 46 | 1.00 | **-** | **-** | **-** | 1.00 | **-** | **-** | **-** |
| overweight age 26/29 | 1.94 | 0.93 | 4.06 | 0.079 | 1.84 | 0.86 | 3.93 | 0.12 |
| overweight age 34/42 | 1.75 | 0.71 | 4.30 | 0.22 | 1.72 | 0.70 | 4.26 | 0.24 |
| **Contacted NHS 111/24hr (n=2,720) – Reference: no NHS contact** | | | | | | | | |
| Reference: Never Overweight | 1.00 | **-** | **-** | **-** | 1.00 | **-** | **-** | **-** |
| overweight age 26 | 1.61 | 1.01 | 2.56 | 0.044 | 1.68 | 1.05 | 2.71 | 0.032 |
| overweight age 29 | 1.55 | 0.88 | 2.73 | 0.13 | 1.62 | 0.91 | 2.87 | 0.10 |
| overweight age 34 | 1.26 | 0.66 | 2.39 | 0.49 | 1.33 | 0.69 | 2.55 | 0.39 |
| overweight age 42 | 1.61 | 0.86 | 3.02 | 0.14 | 1.62 | 0.86 | 3.04 | 0.14 |
| overweight age 46 | 1.53 | 0.79 | 2.97 | 0.21 | 1.48 | 0.76 | 2.88 | 0.25 |
| **Long COVID (n= 876) – Reference: acute COVID** | | | | | | | | |
| Reference: Never Overweight/ Overweight 46 | 1.00 | **-** | **-** | **-** | 1.00 | **-** | **-** | **-** |
| overweight age 26/29 | 1.23 | 0.78 | 1.93 | 0.37 | 1.33 | 0.83 | 2.14 | 0.24 |
| overweight age 34/42 | 1.16 | 0.64 | 2.10 | 0.62 | 1.20 | 0.65 | 2.22 | 0.56 |

**Table S4. Footnote:** Multiply imputed logistic regression results for associations between age first overweight and COVID-19 outcomes in NCDS and BCS70 without use of COVID-19 and IP weights. In NCDS, “never overweight” is the reference category. In BCS70, “Never Overweight/First Overweight 46” is the reference category.

# Results S1. Sensitivity Analysis: unweighted analysis

The sensitivity analysis without COVID-19 and IP weights differed slightly from the main analysis, especially for results regarding age of first overweight. In addition to associations seen for first overweight at age 23 and 33 in NCDS, associations were also observed for BMI at age 42 with self-reported COVID-19 infection. Associations were additionally observed for overweight at 42/44 and 50/55 and contacting NHS111 whilst no associations were observed in the weighted analysis. For BCS70, associations were observed in unweighted analysis between first age of overweight at ages 26, 29, 34 and 46 with self-reported COVID-19 illness, whilst none were observed in the main analysis.

For age first obese in NCDS, results were similar in the sensitivity analysis to those reported in the main analysis, except for an additional association between first being obese at age 42/44 and antibody confirmed COVID-19 illness, not observed in the main analysis. For BCS70, additional association were observed between age of first obesity at 26 and 42 and self-reported COVID-19 infection, and obesity at age 26 and 42 with contacting NHS111, not present in the weighted analysis.
